# LncRNA *TUG1* Promotes Apoptosis, Invasion, and Angiogenesis of Retinal Endothelial Cells in Retinopathy of Prematurity *via* MiR-145-5p

**DOI:** 10.3389/fmed.2022.803214

**Published:** 2022-04-04

**Authors:** Yuexia Wang, Yue Wang, Xue Wang, Yuan Ma, Zhaojin Li, Yu Di

**Affiliations:** Shengjing Hospital of China Medical University, Shenyang, China

**Keywords:** long non-coding RNA taurine up-regulated gene 1 (*TUG1*), miR-145-5p, cellular communication network factor 1 (CCN1), human retinal endothelial cells (HRECs), retinopathy of prematurity (ROP), retinal neovascularization (RNV)

## Abstract

**Purpose:**

Retinopathy of prematurity (ROP) is a common retinal vascular disease in premature neonates. In recent years, there is increasing evidence that the long non-coding RNA taurine upregulated gene 1 (*TUG1*) plays a regulatory role in vascular diseases, suggesting a potential role for *TUG1* in vascular endothelial cells. We hypothesized that *TUG1* may be associated with ROP. Our aim, therefore, was to explore the biological functions of *TUG1* in aberrant retinal development.

**Methods:**

We used the mouse oxygen-induced retinopathy (OIR) model to simulate the pathological changes of retinal in ROP. Quantitative real-time polymerase chain reaction was used to detect the expression of *TUG1*, miR-145-5p and cellular communication network factor 1 (CCN1). Human retinal endothelial cells (HRECs) were treated with CoCl_2_ to mimic hypoxia conditions. Cellular functional changes were observed after transfection with RNA interference (RNAi)-TUG1 and miR-145-5p mimics. The apoptosis of HRECs was detected by flow cytometry, the migration ability was detected by wound healing and transwell migration assays, and the ability of angiogenesis was detected by tube formation assay. The potential binding sites between *TUG1*, miR-145-5p, and CCN1 were verified by dual-luciferase reporter assays. The degree of retinopathy was evaluated by staining retinal sections with hematoxylin and eosin, and the expression of CCN1, HIF-1α, VEGF, caspase-3, Bcl-2, IL-1β, and TNF-α protein was analyzed by Western blotting and immunohistochemistry.

**Results:**

In the retina tissue of OIR mice, *TUG1*, miR-145-5p, and CCN1 were differentially expressed. Knocking down *TUG1* attenuated apoptosis, migration, and angiogenesis induced by hypoxia on HRECs, as did miR-145-5p overexpression. Results from reporter assays indicate direct interactions between *TUG1*, miR-145-5p, and CCN1. Intravitreal injection of miR-145-5p mimics reduced the degree of retinopathy.

**Conclusion:**

*TUG1* acts as a molecular sponge of miR-145-5p to regulate CCN1 expression and thus regulate the development of retinal neovascularization. This regulatory mechanism may provide a new theoretical basis for the prevention and treatment of ROP.

## Introduction

Retinopathy of prematurity (ROP) is a retinal vascular disease that occurs in preterm and low birth weight infants. While the details of the pathogenic process are not clear, it is believed that the formation of retinal non-perfusion areas and pathological retinal neovascularization (RNV) lead to ROP (1, 2). At present, retinal laser photocoagulation and vitreous injection of anti-vascular endothelial growth factor (VEGF) drugs are commonly used to control the development of ROP. However, laser photocoagulation may damage the retina, and drugs injected into the vitreous cavity may enter the blood circulation, adversely affecting the underdeveloped organ systems of premature infants. Even if drugs are combined with laser therapy, there are serious adverse reactions (3–5). Therefore, at present, anti-VEGF drugs are still used cautiously in the treatment of ROP. In light of these drawbacks, the prevention and treatment of ROP is still an unsolved problem, and there is an urgent need to identify new strategies to combat this disease.

Long non-coding RNAs (lncRNAs) are a class of RNA transcripts that are greater than 200 bp in length and have almost no potential to encode proteins (6). The lncRNA taurine upregulated gene 1 (*TUG1*) was discovered relatively early in the history of lncRNA investigations, and it is indispensable in the development of the retina (7). It has also been implicated in various disease-linked cellular processes, including cell proliferation, cell differentiation, cell invasion, radiation resistance, angiogenesis, and inflammation (8, 9). In particular, it plays an important role in neovascularization and the malignant degree and prognosis of many tumor types (10). It plays an important role in biological regulation by acting as a competitive endogenous RNA (or sponge) for microRNAs (miRNAs), thereby regulating downstream mRNAs (11). The miRNAs that bind to *TUG1* include miR-1299, miR-186-5p, miR-212-3p, miR-145, miR-26a, and so on (12–14). MiRNAs are endogenous non-coding small RNAs that are 21–23 nucleotides in length and highly conserved in evolution (15, 16). LncRNAs can compete with miRNAs to regulate the proliferation and migration of human retinal endothelial cells (HRECs) and the formation of RNV in diabetic retinopathy (17). Based on the fact that the molecular mechanism of RNV in ROP is similar to that of diabetic retinopathy, we speculated that lncRNAs and miRNAs may also be involved in the regulation of ROP. Cellular communication network factor 1 (CCN1) is a stromal cell protein involved in vascular development and tissue repair. In our previous study, we found that CCN1 is highly expressed during RNV in the formation of ROP (18), and a study that analyzed human clinical specimens also showed that the level of CCN1 increased in some ocular vascular complications (19). Results from bioinformatics analyses (*via* the starBase database 2.0.^1^) (20) predict that miR-145-5p is a target of *TUG1* and CCN1 and suggest that *TUG1* may affect the expression of CCN1 by regulating miR-145-5p. Therefore, this study aimed to investigate the expression and function of the lncRNA *TUG1* and miR-145-5p in a mouse model of oxygen-induced retinopathy (OIR) and HRECs. Our results provide new insights for clinical investigations focused on improving treatments for ROP.

## Materials and Methods

### Animals and Oxygen-Induced Retinopathy Model

All animal experiments were performed according to the ARRIVE guidelines and in accordance with the EU Directive 2010/63/EU for animal experiments. These experiments also complied with the Chinese Guidelines for Ethical Review of Laboratory Animal Welfare (GB/T 35892-20181). The Medical Ethics Committee of Shengjing Hospital of China Medical University approved the use of animals for the experiments (ethics code: 2018PS239K) on 1 March 2018. C57BL/6J mice were purchased from Shenyang Changsheng Biological Technology Co., Ltd. [Shenyang, China; license No.: SCXK (Liao) 2015-0001]. Mice were reared in the standard pathogen-free facility for experimental animals at the Benxi base of Shengjing Hospital under temperature conditions of 23 ± 2°C, with a 12-h light/dark cycle. The pups were randomly divided into the control, OIR, OIR-negative control (NC), and OIR-mimics groups, with 60 pups per group. An OIR model was established using the classical Smith method (21). The pups in the control groups were placed in normoxia up to postnatal day (PD) 17. On PD 7, pups in OIR, OIR-NC, OIR-mimics groups were placed in a hyperoxia chamber (75 ± 2%) for 5 days and subsequently removed to normoxic condition for a further 5 days up to PD 17. OIR-NC and OIR-mimics groups received intravitreal injection under isoflurane inhalation anesthesia on PD12. The pups in OIR-NC group received intravitreal injection of 0.5 μL of miR-145-5p mimics negative control reagent (miR-145-5p NC mimics) + 0.5 μL of animal infection reagent (IVG2101) (InvivoGene, China) + 0.5 μL of PBS, and the OIR-mimics group was treated with 0.5 μL of miR-145-5p mimics reagent (miR-145-5p mimics) + 0.5 μL of animal infection reagent + 0.5 μL of PBS. The sequences of miR-145-5p NC mimics were as follows: sense: 5′-UUCUCCGAACGUGUCACGUTT-3′; antisense: 5′-ACGUGACACGUUCGGAGAATT-3′. The miR-145-5p mimics sequences were as follows: sense: 5′-GUCCAGUUUUCCCAGGAAUCCCU-3′; antisense: 5′-GGAUUCCUGGGAAAACUGGACUU-3′. The retinal tissue for polymerase chain reaction (PCR) was collected on PD14, and on PD17 for Western blotting and other follow-up experiments.

### Cell Lines and Cell Culture

The HREC and human renal epithelial (HEK293T) cell lines were purchased from American Type Culture Collection (ATCC, Gaithersburg, MD, United States). The cells were cultured in RPMI 1640 medium containing 10% fetal bovine serum (FBS) (Gibco, NY, United States) in a humidified incubator (Thermo Scientific, MA, United States) at 37°C, with 5% CO_2_. To simulate hypoxic conditions, cells were treated with 200 μmol/L CoCl_2_ (Sigma-Aldrich, St. Louis, MO, United States) (22). When knocking down *TUG1*, cell culture experiments comprised the following groups: HRECs cultured under normal conditions (control group), HRECs treated with CoCl_2_ (CoCl_2_ group), HRECs transfected with negative control RNA interference (RNAi) and treated with CoCl_2_ (CoCl_2_ + NC group), and HRECs transfected with TUG1-RNAi and treated with CoCl_2_ (CoCl_2_ + RNAi group). When overexpressing miR-145-5p, cell culture experiments comprised the following groups: control group, CoCl_2_ group, HRECs transfected with miR-145-5p NC mimics and treated with CoCl_2_ (CoCl_2_ + NC group), and HRECs transfected with miR-145-5p mimics and treated with CoCl_2_ (CoCl_2_ + mimics group).

### Transient Transfections

Human retinal endothelial cells were evenly seeded into a six-well plate and transfected at 60–70% confluency. TUG1 RNAi (TUG1-RNAi), negative control TUG1-RNAi (RNAi-NC) (Genechen, Shanghai, China), miR-145-5p mimics (miR-145-5p mimics), and miR-145-5p NC mimics (NC mimics) (GenePharma, Shanghai, China) were transfected into the cells using the liposome transfection reagent Lipofectamine 3000 (Invitrogen, Waltham, MA, United States), according to the manufacturer’s instructions. Before the experiment, we examined the transfection efficiency of the plasmids and mimics by quantitative real-time PCR (qRT-PCR). The sequences of TUG1-RNAi, RNAi-NC, miR-145-5p mimics, and NC mimics are as follows:

TUG1-RNAi 1: 5′-CGAGATGATTCCTACCACCTT-3′; TUG1-RNAi 2: 5′-GCCAAATTCAAATACTGGCAA-3′; TUG1-RNAi 3: 5′-CCTCCATGAATACCTGAATTA-3′; RNAi-NC: 5′-TTCTCCGAACGTGTCACGT-3′; miR-145-5p mimics: sense: 5′-GUCCAGUUUUCCCAGGAAUCCCU-3′; miR-145-5p mimics: antisense: 5′-GGAUUCCUGGGAAAACUGGACUU-3′; NC mimics: sense: 5′-UUCUCCGAACGUGUCACGUTT-3′; NC mimics: antisense: 5′-ACGUGACACGUUCGGAGAATT-3′.

### RNA Extraction and Quantitative Real-Time Polymerase Chain Reaction

Total RNA was extracted from cultured cells and retina tissue using TRIzol (Takara Biotechnology, Otsu, Japan), and the expression of *TUG1*, miRNA-145-5p, and *CCN1* mRNA was detected by qRT-PCR. cDNA was synthesized using the Mir-X miRNA First-Strand Synthesis kit (Cat. No. 638313, Takara Biotechnology, Otsu, Japan) and the PrimeScript RT reagent kit with gDNA Eraser (Code No. RR047Q, Takara Biotechnology). PCR was then performed on a 7500 Fast Real-Time PCR machine (Applied Biosystems, Warrington, United Kingdom) using TB Green^®^ Premix Ex Taq™ II (Tli RNaseH Plus, code no. RR820A, Takara Biotechnology). The reaction conditions were as follows: 40 cycles of denaturation for 30 s at 95°C, amplification for 3 s at 95°C and 30 s at 60°C. β-Actin and U6 were used as internal reference genes for mRNA and miRNA analyses, respectively. For quantitative analysis of differential expression, data were processed using the 2^–ΔΔCt^ method, with the mRNA expression presented as the relative fold change compared to β-actin, and miRNA expression presented as the relative fold change compared to U6. The primers (Shenggong, Shanghai, China) used are shown in Table 1.

**TABLE 1:** Primer sequences used for qRT-PCR.

Gene name	Primer sequence (5′ to 3′)
LncRNA-*Tug1* (mouse)	Forward: 5′-GAGACACGACTCACCAAGCACTG-3′
	Reverse: 5′-CAGAAGGAAGGTCATTGGCAGGTC-3′
LncRNA-*TUG1* (human)	Forward: 5′-GCAAGCACTACCACCAGCACTG-3′
	Reverse: 5′-CACTCAGCAATCAGGAGGCACAG-3′
MiR-145-5p (human/mouse)	Forward: 5′-CCGGTCCAGTTTTCCCAGGAATCCCT-3′
CCN1 (human)	Forward: 5′-CTTGTGAAAGAAACCCGGATTT-3′
	Reverse: 5′-ACTCAAACATCCAGCGTAAGTA-3′
CCN1 (mouse)	Forward: 5′-CCCAGAACCAGTCAGATTTACT-3′
	Reverse: 5′-GAAAACATCCTCTCCATCTTCGC-3′
β-Actin (mouse)	Forward: 5′-GTGCTATGTTGCTCTAGACTTCG-3′
	Reverse: 5′-ATGCCACAGGATTCCATACC-3′
β-Actin (human)	Forward: 5′-CCTGGCACCCAGCACAAT-3′
	Reverse: 5′-GGGCCGGACTCGTCATAC-3′
U6	Forward: 5′-ACAGATCTGTCGGTGTGGCAC-3′
	Reverse: 5′-GGCCCCGGATTATCCGACATTC-3′

### Flow Cytometry for Apoptosis

Twenty-four hours after transfection, HRECs were treated with 200 μmol/L CoCl_2_ for 24 h. Then, the cells were collected by treating with trypsin without EDTA and subjected to AnnexinV-PE/7-AAD labeling using an apoptosis detection kit (Vazyme, Nanjing, China). The cells were incubated at room temperature and protected from light for 10 min according to the manufacturer’s instructions and analyzed by flow cytometry (BD, FACSCalibur, NJ, United States). Cell Quest Pro software (Becton-Dickinson, CA, United States) was used for data analysis.

### Wound Healing Assay

Human retinal endothelial cells were uniformly seeded into a six-well culture plate, and three horizontal lines were marked on the bottom of each well. The cells were transfected and treated with CoCl_2_ as described earlier. When the cells reached a confluency of approximately 90%, the tip of a 10 μL micropipette was used to make a uniform scratch through the cell monolayer, perpendicular to the horizontal line. The disrupted cell mass was gently washed off with PBS, and the remaining adherent cells were incubated in a serum-free culture medium. After 48 h, wound healing was examined and photographed.

### Transwell Migration Assay

The cells were transfected and treated with CoCl_2_ as described earlier. Next, 3 × 10^4^ cells were suspended in 200 μL of serum-free RPMI1640 medium and transferred to the upper chamber of the transwell insert in a 24-well plate (Corning, NY, United States), and 500 μL of medium containing 10% FBS was added to the lower chamber. The cells were incubated at 37°C for 24 h, after which the remaining cells in the upper chamber were gently scraped off with cotton swabs. The transwell membrane was then fixed with 4% paraformaldehyde for 20 min, washed with PBS, stained with 0.5% hematoxylin solution for 20 min, and washed again with PBS. Finally, we analyzed the number of cells on the bottom of the membrane using an inverted microscope (ECLIPSE Ni-U, Nikon, Tokyo, Japan). For each well, the average number of cells was calculated from five randomly selected fields of view.

### Tube Formation Assay

The bottom of a 96-well plate was evenly coated with 50 μL of Matrigel (Corning, NY, United States) diluted 2:1 in a basic medium and incubated at 37°C for 30 min to form a gel. The cells were suspended in a medium containing 10% FBS, and 1 × 10^4^ cells were seeded per well in the Matrigel-coated 96-well plate. After 6 h of incubation, each well was examined and photographed using an inverted microscope (ECLIPSE Ni-U, Nikon). Each treatment group comprised three wells, and 1–2 fields of view were captured for each well. The overall experiment was repeated five times. In each field of view, we counted the number of nodes and meshes and measured the total branching length of the tubes.

### Dual-Luciferase Reporter Assays

Dual luciferase reporter assays were used to verify binding of miR-145-5p with *TUG1* and CCN1. The sites in miR-145-5p that facilitate binding with TUG1 and CCN1 were predicted *via* the starBase database. An expression vector was designed and constructed. First, 5 × 10^3^ HEK293T cells were seeded into each well in a 96-well plate. After 12 h, 3 pmol miR-145-5p mimics or miR-145-5p NC mimics were co-transfected with 0.1 μg TUG1-WT or TUG1-MUT using Lipofectamine 3000, according to the manufacturer’s instructions. After a further 36 h, the cells were lysed with 1 × passive lysis buffer from the Dual-Luciferase Reporter Assay System (Promega, WI, United States), and luciferase activity was measured on a luminometer (Synergy H1 microplate reader, BioTek, VT, United States). Firefly luciferase activity was normalized to Renilla luciferase activity. Each transfection reaction was performed in triplicate. The same procedure was used for the co-transfection of miR-145-5p mimics or miR-145-5p NC mimics with CCN1-WT or CCN1-MUT.

### Retinal Vascular Staining

Under isoflurane inhalation anesthesia, eyeballs from 17-day-old mice were removed and fixed in 4% paraformaldehyde overnight. The limbus cornea was incised and the anterior segment and vitreous body were removed. Retinal tissue was separated and cleaned with PBS, the Isolectin IB4 Alexa Fluor^®^ dye conjugate (Invitrogen, United States) was dissolved in PBS solution containing 1% TritonX-100 (Solarbio, Beijing, China), and incubated with the retinal tissue at 4°C overnight. The retinal tissue was taken out the next day, cleaned with PBS, placed on glass slides, and cut into four sections under the microscope. The slices were sealed with an anti-quenching agent and photographed with a fluorescence microscope (ECLIPSE Ni-U, Nikon). ImageJ software 1.51 (NIH, United States) was used to calculate the retinal avascular area.

Detailed steps are as follows:

1. Open the picture, click Image → Type → 8-bit.
2. Select the heart-shaped circle, the Freehand Selections are displayed.
3. Circle the entire area of the retina in the picture with the mouse, click Analyze → Measurement, to get the total area of the retina.
4. Re-select the non-perfusion area of the retina and click Analyze → Measurement, to obtain the non-perfusion area of the retina.
5. Enlarge the picture and circle the neovascularization part of the retina as much as possible to get the neovascularization area of the retina.
6. Non-perfusion area/Total retinal area = percentage of areas without vascular perfusion; Neovascularization area/Total retinal area = percentage area of neovascularization.

### Hematoxylin and Eosin Staining

The eyeballs of 17-day-old mice were fixed in 4% paraformaldehyde overnight, embedded in paraffin, and cut into 4 μm thick sections. The slices were dried at 60°C for 6, dewaxed, stained with hematoxylin and eosin (HE), dehydrated, and sealed with neutral gum. The number of nuclei penetrating the inner limiting membrane of the retina was directly counted by the double-blind method.

### Immunohistochemistry

Immunohistochemistry was performed with the Streptavidin-Biotin Complex (SABC) kit (Boster Bio, Pleasanton, CA, United States). The paraffin sections were first dewaxed and thermally repaired with a 3% citric acid repair solution. Then, 3% H_2_O_2_ was added dropwise and incubated at room temperature for 10 min. The sections were washed three times with PBS and blocked with goat serum at 37°C for 30 min. Then, the sections were incubated with diluted primary antibody [CCN1, hypoxia-inducible factor-1 α (HIF-1α), VEGF, caspase-3, Bcl-2, IL-1β, and TNF-α, 1:200, ImmunoWay, Plano, TX, United States] at 4°C overnight. On the second day, the sections were washed with PBS three times. Biotinylated goat anti-rabbit IgG was dropped and incubated at 37°C for 30 min, then washed with PBS three times. The SBC-POD reagent was added dropwise to the samples and incubated at 37°C for 30 min. For negative control groups, the primary antibody was replaced with PBS. Under the microscope, 3, 3′-diaminobenzidine was applied for 50 s and counterstained with hematoxylin, sealed with neutral gum, and then observed and photographed under the microscope. Brown cells were positive for the respective antibody, and ImageJ was used to calculate the cumulative optical density of each immunohistochemical section by the double-blind method.

### Western Blotting

The treated cells and retinal tissue were lysed in RIPA lysis buffer (Solarbio) containing 1% PMSF (Solarbio), and the protein concentration was determined using BCA assay (BCA Protein Quantification Kit, Vazyme, Nanjing, China). Then, protein samples were separated with a 10% fast electrophoresis gel preparation kit (EpiZyme, Shanghai, China) and transferred to a PVDF membrane (Merck Millipore, Burlington, MA, United States). After blocking with 5% bovine serum albumin for 1 h, membranes were incubated in primary antibody overnight at 4°C. The primary antibodies used were rabbit-derived anti-CCN1 (1:1,000) and rabbit-derived anti-tubulin (1:1,000) (both Immunoway). The secondary antibody was horseradish peroxidase-labeled goat anti-rabbit IgG (H + L) (1:1,000, Immunoway). After washing with TBST (0.05 M Tris, 0.15 M NaCl, pH 7.4 with 0.1% Tween-20), the PVDF membrane was developed on an Amersham Imager 680 with chemiluminescent reagents (BeyoECL Plus, Biyuntian, Shanghai, China). ImageJ software 1.51 (NIH) was used to normalize the intensity of the target protein signal to that of tubulin.

### Statistical Analysis

All analyses were performed in GraphPad Prism 8.0 software (GraphPad Software Inc., CA, United States). All values are presented as the mean ± standard deviation (SD) of three or more independent experiments. Statistical analysis was performed using Student’s *t*-test or one-way ANOVA. *P < 0.05, **P < 0.01, and ***P < 0.001 indicate significant differences.

## Results

### *Tug1* Expression Was Upregulated and MiR-145-5p Was Down-Regulated in Oxygen-Induced Retinopathy Mice

**FIGURE 1:**
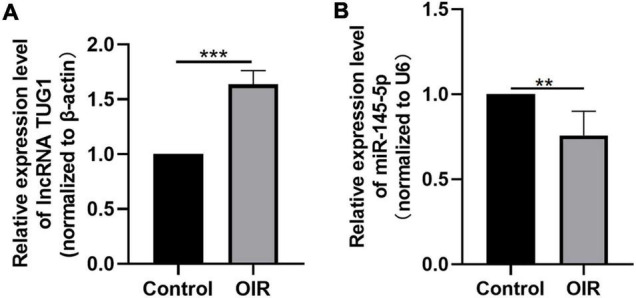
*Tug1* expression was upregulated and miR-145-5p was down-regulated in OIR mice. **(A)** *Tug1* expression level (mean ± SD, *n* = 5). ***P < 0.001. **(B)** MiR-145-5p expression level (mean ± SD, *n* = 5). **P < 0.01.

We analyzed the expression of *Tug1* and miR-145-5p in the control group and the OIR group by using qRT-PCR. The results showed that the expression of *Tug1* in the OIR group was significantly higher than that in the control group (Figure 1A, ***P < 0.001), while the expression of miR-145-5p significantly lower (Figure 1B, **P < 0.01).

### Knocking Down Taurine Upregulated Gene 1 Inhibits Apoptosis, Migration, and Angiogenesis in Human Retinal Endothelial Cells Induced by Hypoxia

**FIGURE 2:**
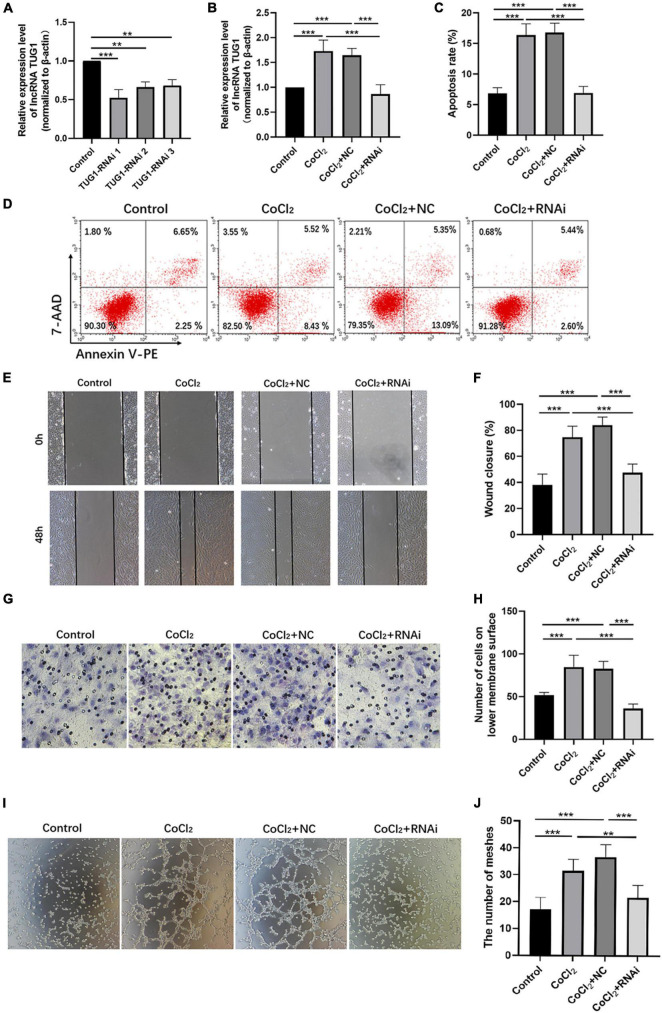
Knocking down *Tug1* inhibits apoptosis, migration, and angiogenesis in HRECs induced by hypoxia. **(A)** The transfection efficiency of TUG1-RNAi (mean ± SD, *n* = 3). ***P < 0.001, **P < 0.01. **(B)** *TUG1* expression level (mean ± SD, *n* = 5). ***P < 0.001. **(C)** Flow cytometry results showing the percentage of apoptotic cells (mean ± SD, *n* = 5). ***P < 0.001. **(D)** Results of flow cytometry with cells labeled with Annexin-PE and 7-Aminoactinomycin D (7-AAD). **(E)** Results of wound healing assay, 100× magnification. **(F)** Quantitation of results from the wound healing assay in **(E)** (mean ± SD, *n* = 5). ***P < 0.001. **(G)** Results of transwell migration assay, 200× magnification. **(H)** Quantitation of results from the transwell migration assay in **(G)** (mean ± SD, *n* = 5). ***P < 0.001. **(I)** Results of tube formation assay, 100× magnification. **(J)** Comparison of the number of meshes in different treatment groups (mean ± SD, *n* = 5), ***P < 0.001, *P < 0.05.

As shown in Figure 2A, *TUG1* expression was successfully knocked down in normal HRECs when transfected with TUG1-RNAi, and TUG1-RNAi 1 was selected for subsequent experiments. Next, we determined the relative expression of *TUG1* in the control, CoCl_2_, CoCl_2_ + NC, and CoCl_2_ + RNAi groups (Figure 2B). *TUG1* expression was significantly higher in the CoCl_2_ group compared to the control (***P < 0.001), and expression of the *TUG1* was significantly lower in the CoCl_2_ + RNAi group compared to the CoCl_2_ + NC (***P < 0.001). These results suggest that TUG1-RNAi successfully inhibited the increase in *TUG1* induced by hypoxia. Flow cytometry analysis indicated that the apoptosis was increased in the CoCl_2_ group compared with the control (Figures 2C,D, ***P < 0.001) and was significantly inhibited in the CoCl_2_ + RNAi group compared with the CoCl_2_ + NC (***P < 0.001). The wound healing and transwell migration assays showed that treatment with CoCl_2_ promoted HREC migration compared with that in the control (Figures 2E–H, wound healing assay***P < 0.001, transwell assay***P < 0.001), the migratory ability of HRECs in the CoCl_2_ + RNAi group was lower than that in the CoCl_2_ + NC group (wound healing assay***P < 0.001, transwell migration assay***P < 0.001). Together, these data indicate that hypoxia induced by CoCl_2_ promoted HREC migration and knocking down *TUG1* inhibited the migratory ability of these cells. The formation of RNV is related to the tube-forming ability of endothelial cells, and therefore, we examined the angiogenic activity of HRECs using a tube formation assay. The results demonstrated that, compared with the control group (Figures 2I,J), the CoCl_2_ group exhibited a greater number of meshes (***P < 0.001), which promoted tube formation in the HRECs. Compared with the CoCl_2_ + NC group, the CoCl_2_ + RNAi group exhibited fewer meshes (***P < 0.001), indicating that loss of *TUG1* reduces the angiogenic capacity of HRECs.

### MiR-145-5p Interacts With Taurine Upregulated Gene 1 and Cellular Communication Network Factor 1

**FIGURE 3:**
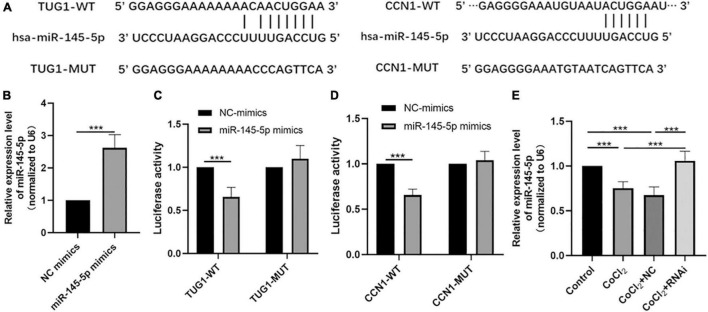
MiR-145-5p interacts with *TUG1* and CCN1. **(A)** The predicted binding sites between miR-145-5p and TUG1, and those between miR-145-5p and CCN1 from bioinformatic analysis using starBase interface. **(B)** The transfection efficiency and expression of miR-145-5p (mean ± SD, *n* = 5). ***P < 0.001. **(C)** Luciferase reporter assays of TUG1 and miR-145-5p (mean ± SD, *n* = 5). ***P < 0.001. **(D)** Luciferase reporter assays of miR-145-5p and CCN1 (mean ± SD, *n* = 5). ***P < 0.001. **(E)** MiR-145-5p expression level (mean ± SD, *n* = 5). ***P < 0.001.

Bioinformatic analyses using the starBase interface indicated potential binding sites between *TUG1* and miR-145-5p (Figure 3A). The transfection efficiency of miR-145-5p mimics was confirmed by qRT-PCR (Figure 3B, ***P < 0.001). Results from luciferase reporter assays showed that in the TUG1-WT group, transfection with miR-145-5p mimics significantly reduced relative luciferase activity compared with that of the NC mimics (Figure 3C, ***P < 0.001), suggesting an interaction between them.

Our bioinformatic analyses also predicted the presence of potential binding sites between miR-145-5p and CCN1 (Figure 3A). The results of luciferase reporter assays also indicated a direct binding interaction between CCN1 and miR-145-5p (Figure 3D, ***P < 0.001). We next examined the expression of miR-145-5p in HRECs in the control, CoCl_2_, CoCl_2_ + NC, and CoCl_2_ + RNAi groups using qRT-PCR (Figure 3E). As expected, the expression of miR-145-5p in the CoCl_2_ group was lower than that in the control group (***P < 0.001), while miR-145-5p expression was higher in the CoCl_2_ + RNAi compared to that in the CoCl_2_ + NC group (***P < 0.001). These results suggest that changes in *TUG1* expression can produce corresponding changes in miR-145-5p levels.

### Overexpressing MiR-145-5p Inhibits Apoptosis, Migration, and Angiogenesis in Human Retinal Endothelial Cells Induced by Hypoxia

**FIGURE 4:**
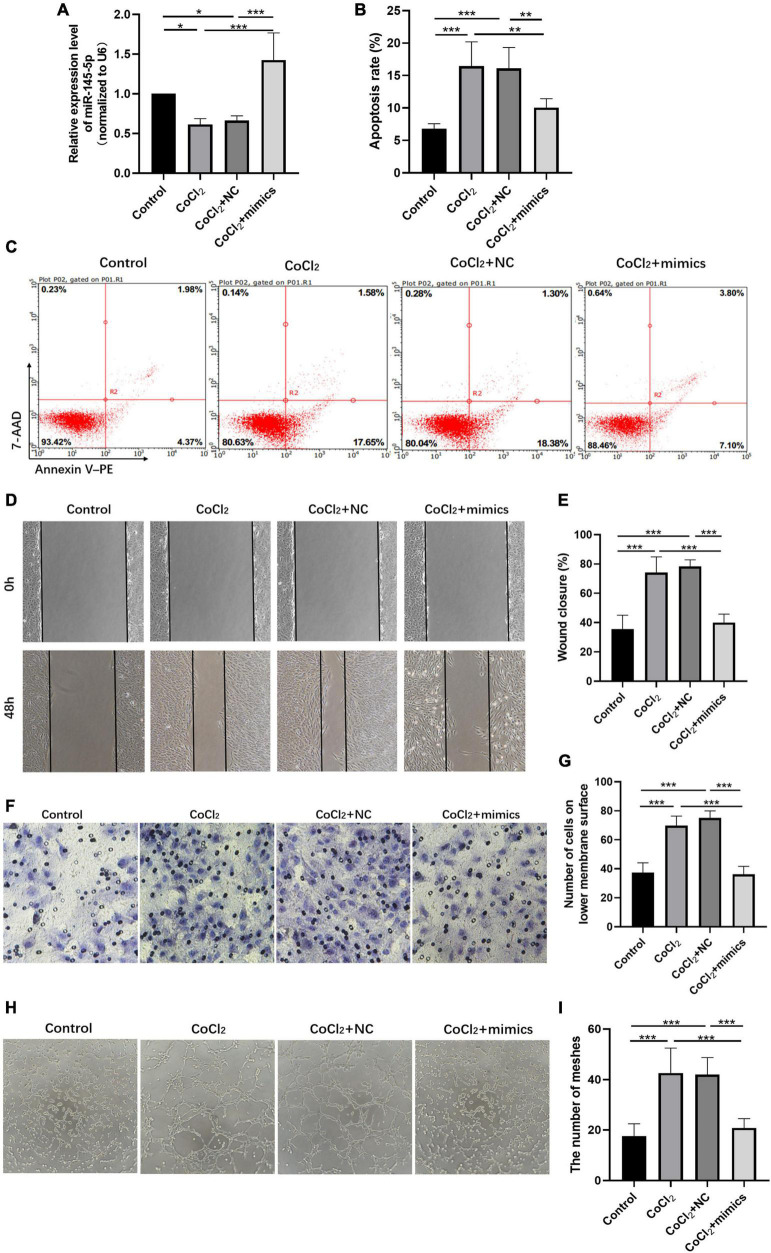
Overexpressing miR-145-5p inhibits apoptosis, migration, and angiogenesis in HRECs induced by hypoxia. **(A)** MiR-145-5p expression level (mean ± SD, *n* = 5). *P < 0.05. **(B)** Flow cytometry results showing the percentage of apoptotic cells (mean ± SD, *n* = 5). ***P < 0.001. **(C)** Results of flow cytometry with cells labeled with Annexin-PE and 7-Aminoactinomycin D (7-AAD). **(D)** Results of the wound healing assay, 100× magnification. **(E)** Quantitation of results from the wound healing assay in **(D)** (mean ± SD, *n* = 5). ***P < 0.001. **(F)** Results of transwell migration assay, 200× magnification. **(G)** Quantitation of results from the transwell migration assay in **(F)** (mean ± SD, *n* = 5). ***P < 0.001. **(H)** Results of tube formation assay, 100× magnification. **(I)** Comparison of the number of meshes in different treatment groups (mean ± SD, *n* = 5), ***P < 0.001, **P < 0.01.

We further explored the influence of miR-145-5p on the biological functions of HRECs. The relative expression of miR-145-5p in the control, CoCl_2_, CoCl_2_ + NC, and CoCl_2_ + mimics groups was determined using qRT-PCR analysis (Figure 4A). The results showed that miR-145-5p expression decreased in the CoCl_2_ group compared to the control group (*P < 0.05) but increased in the CoCl_2_ + mimics group compared to the CoCl_2_ + NC group (***P < 0.001). The percentage of apoptotic cells was reduced in the CoCl_2_ + mimics group compared with the CoCl_2_ + NC group (Figures 4B,C, ***P < 0.001), suggesting that the overexpression of miR-145-5p inhibits apoptosis. The results from the wound healing and transwell migration assays revealed that the migration rate of the CoCl_2_ + mimics group was lower than that of the CoCl_2_ + NC group (Figures 4D–G, wound healing assay ***P < 0.001, transwell migration assay***P < 0.001), supporting the role of overexpression of miR-145-5p in inhibiting cell migration. The results from the tube formation assay revealed a similar trend (Figures 4H,I). Compared with the CoCl_2_ + NC group, the CoCl_2_ + mimics group exhibited fewer meshes (***P < 0.001).

### Knocking Down Taurine Upregulated Gene 1 and Overexpressing MiR-145-5p Inhibit Cellular Communication Network Factor 1 Expression

**FIGURE 5:**
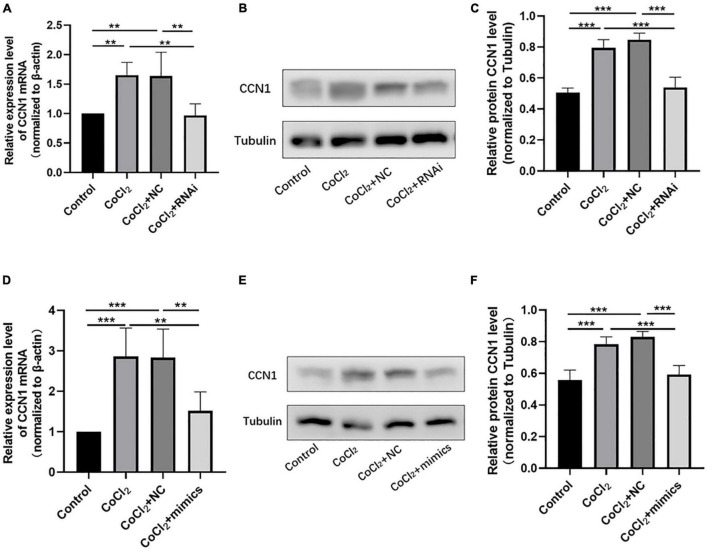
Knocking down *TUG1* and overexpressing miR-145-5p inhibits CCN1 expression. **(A)** CCN1 mRNA expression level (mean ± SD, *n* = 5). **P < 0.01. **(B)** Western blot results of CCN1 expression. **(C)** Quantitative analysis of band intensities from Western blots shown in **(B)** (mean ± SD, *n* = 5). ***P < 0.001. **(D)** CCN1 mRNA expression level (mean ± SD, *n* = 5). ***P < 0.001, **P < 0.01. **(E)** Western blot results of CCN1 expression. **(F)** Quantitative analysis of band intensities from Western blots shown in **(E)** (mean ± SD, *n* = 5). ***P < 0.001.

The qRT-PCR results showed that the expression of CCN1 mRNA in the CoCl_2_ + RNAi group was significantly lower than that in the CoCl_2_ + NC group (Figure 5A, **P < 0.01), the Western blot results showed decreased expression of CCN1 in the CoCl_2_ + RNAi compared to the CoCl_2_ + NC group (Figures 5B,C, ***P < 0.001). Similarly, overexpression of miR-145-5p effectively reduced CCN1 mRNA and protein levels (Figures 5D–F, qRT-PCR ***P < 0.001, **P < 0.01, Western blot ***P < 0.001).

### Overexpression of MiR-145-5p Can Reduce the Degree of Oxygen-Induced Retinopathy Retinal Lesions

**FIGURE 6:**
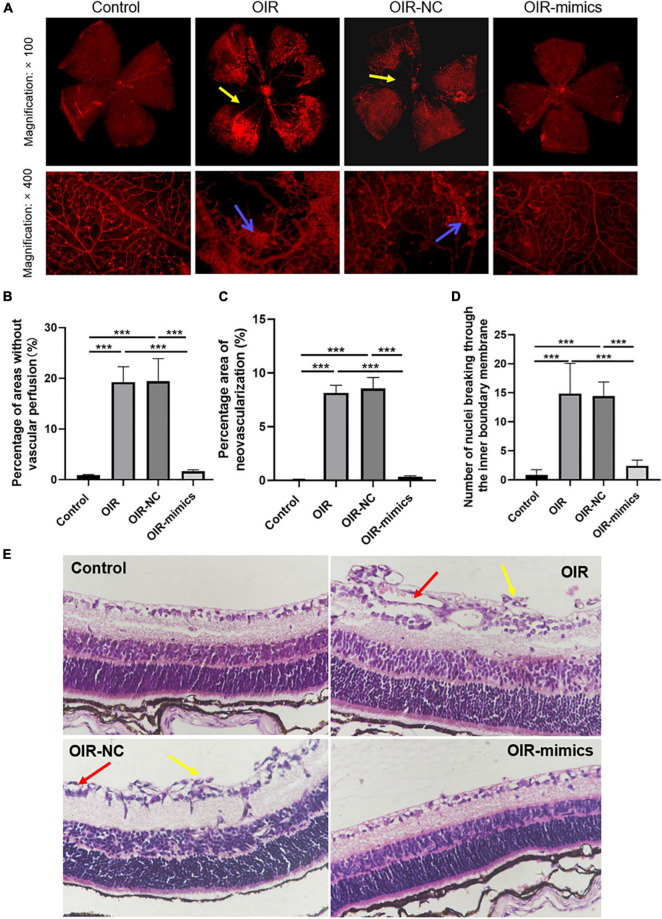
Overexpression of miR-145-5p can reduce the degree of OIR retinal lesions. **(A)** Retinal vascular morphology of mice in different treatment groups, yellow arrows indicate non-perfusion area, blue arrows indicate neovascularization. **(B)** Percentage of areas without vascular perfusion (mean ± SD, *n* = 7). ***P < 0.001. **(C)** Percentage area of neovascularization (mean ± SD, *n* = 7), ***P < 0.001. **(D)** Number of nuclei breaking through the inner limiting membrane (mean ± SD, *n* = 7), ***P < 0.001. **(E)** HE staining images in different treatment groups, yellow arrows indicate nuclei breaking through the inner limiting membrane, red arrows indicate neovascularization.

Retinal images of 17-day-old mice are shown in Figure 6A. Compared with the control group, the OIR group had a larger non-perfusion area and more neovascularization, while the OIR-mimics group injected with miR-145-5p mimics had a significantly lower non-perfusion area and neovascularization than the OIR-NC group (Figures 6A–C, ***P < 0.001).

Hematoxylin and eosin staining revealed that the retinal cells in the control group were arranged neatly, while those in the OIR group were disorderly, and the number of endothelial nuclei breaking through the inner limiting membrane into the vitreous lumen was significantly increased (Figures 6D,E, ***P < 0.001). Compared with the OIR-NC group, the endothelial cells in the OIR-mimics group were arranged neatly and the number of endothelial nuclei breaking through the inner boundary membrane decreased significantly (***P < 0.001). These results are consistent with our observations of HREC overexpressing miR-145-5p that exhibited reduced migration and angiogenesis capacity. From outside to inside, the retina is as follows: pigment epithelium, optic rod cone layer, outer limiting membrane, outer nuclear layer, outer reticular layer, inner nuclear layer, inner reticular layer, ganglion cell layer, nerve fiber layer, and inner limiting membrane. Further inside the inner limiting membrane is the vitreous body.

### Overexpression of MiR-145-5p Inhibits Inflammation and Apoptosis in Oxygen-Induced Retinopathy Retinal Tissue

**FIGURE 7:**
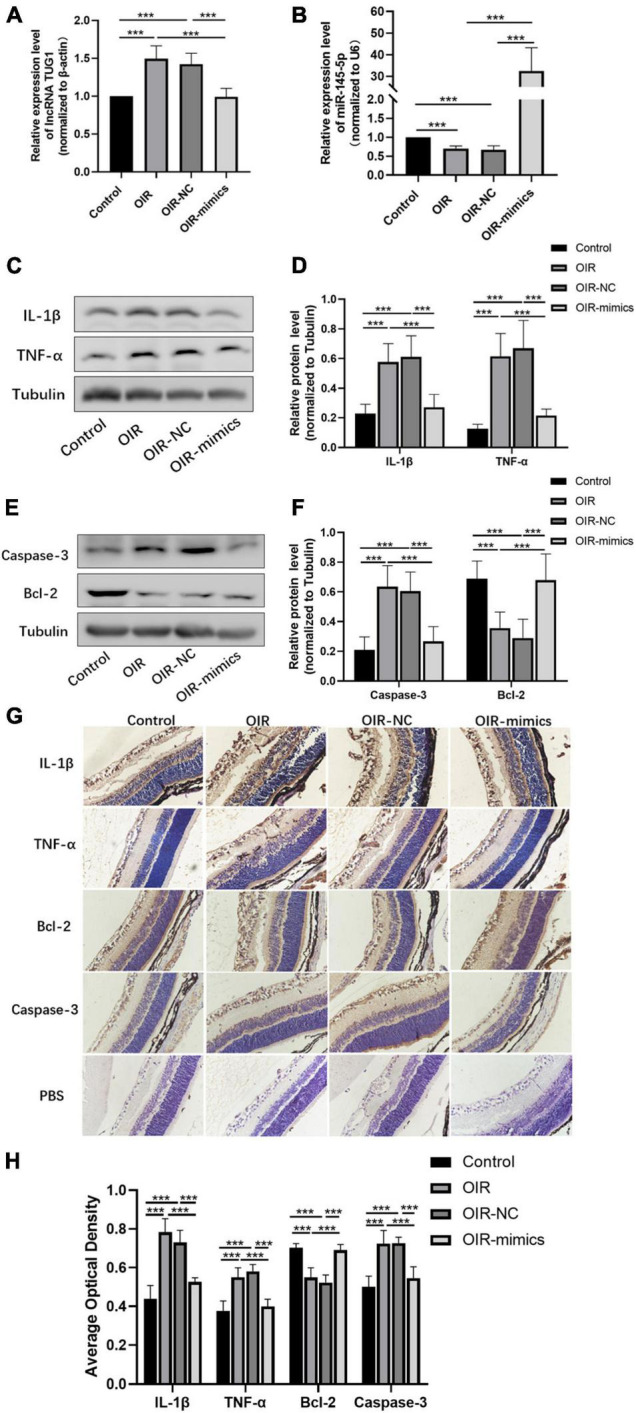
Overexpression of miR-145-5p inhibits inflammation and apoptosis in OIR retinal tissue. **(A)** *Tug1* expression level (mean ± SD, *n* = 6), ***P < 0.001. **(B)** MiR-145-5p expression level (mean ± SD, *n* = 6), ***P < 0.001. **(C)** Western blot results of IL-1β, and TNF-α expression. **(D)** Quantitative analysis of band intensities in **(C)** (mean ± SD, *n* = 7). ***P < 0.001. **(E)** Western blot results of caspase-3, and Bcl-2 expression. **(F)** Quantitative analysis of band intensities in **(E)** (mean ± SD, *n* = 7). ***P < 0.001. **(G)** Immunohistochemical map of IL-1β, TNF-α, Bcl-2, and caspase-3 in the retinal tissues of each group. **(H)** The average optical density of the immunohistochemical map in different treatment groups (mean ± SD, *n* = 7). ***P < 0.001.

The expression of *Tug1* and miR-145-5p in retinal tissue was detected by qRT-PCR analysis. The expression of *Tug1* in the OIR-mimics group was significantly lower than that in the OIR-NC group (Figure 7A, ***P < 0.001), while the expression of miR-145-5p significantly higher (Figure 7B, ***P < 0.001). The results of Western blot showed that compared with the control group, the expression levels of IL-1β, TNF-α, and caspase-3 increased significantly in the OIR group, whereas Bcl-2 expression level decreased (Figures 7C–F, ***P < 0.001). Compared with the OIR-NC group, IL-1β, TNF-α, and caspase-3 decreased and Bcl-2 increased in the OIR-mimics group (***P < 0.001). Immunohistochemistry indicated the same trend (Figures 7G,H, ***P < 0.001).

### Over Expression of MiR-145-5p Inhibits the Expression of Cellular Communication Network Factor 1, Hypoxia-Inducible Factor-1 α, and Vascular Endothelial Growth Factor

**FIGURE 8:**
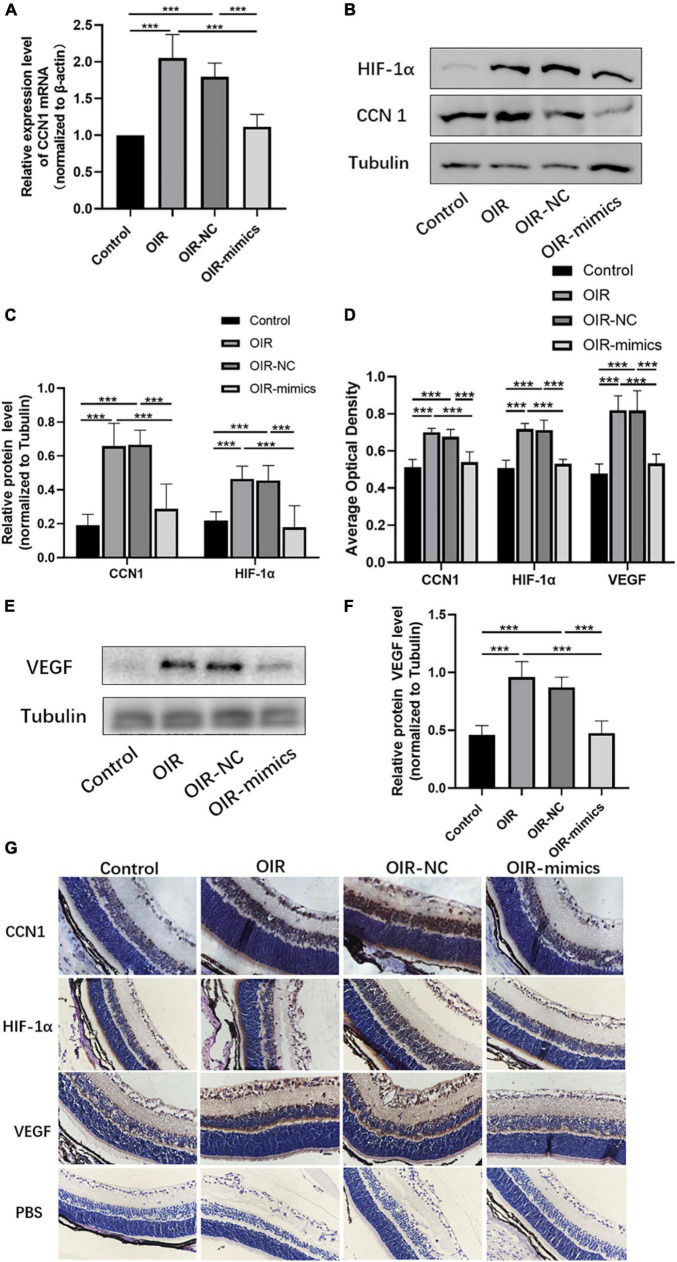
Overexpression of miR-145-5p inhibits the expression of CCN1, HIF-α, and VEGF expression. **(A)** CCN1 mRNA expression level (mean ± SD, *n* = 6), ***P < 0.001. **(B)** Western blot results of CCN1 and HIF-α. **(C)** Quantitative analysis of band intensities from Western blots shown in **(B)** (mean ± SD, *n* = 6). ***P < 0.001. **(D)** The average optical density of the immunohistochemical map in different treatment groups (mean ± SD, *n* = 7). ***P < 0.001. **(E)** Western blot results of VEGF. **(F)** Quantitative analysis of band intensities from Western blots shown in **(E)** (mean ± SD, *n* = 6). ***P < 0.001. **(G)** Immunohistochemical map of CCN1, HIF-α, and VEGF in the retinal tissues.

The qRT-PCR results showed that CCN1 mRNA expression was increased in OIR and miR-145-5p decreased in the OIR-mimics group (Figure 8A, ***P < 0.001). Western blot results showed that the expression of CCN1, HIF-α, and VEGF were significantly increased in the OIR group compared with the control group, while in the OIR-mimics group the same were significantly decreased compared with the OIR-NC group (Figures 8B,C,E,F, ***P < 0.001). Immunohistochemistry also showed the same trend (Figures 8D,G, ***P < 0.001).

## Discussion

The increase in the mean age of mothers and global advances in perinatal medicine have resulted in a high number of premature births and survival, leading to an increased rate of incidence of ROP. The development of ROP is generally believed to be related to the relative hypoxia of the retina. When premature neonates are exposed to supplemental oxygen in incubation chambers, returning them to a normoxic environment can cause vasoconstriction and occlusion, which is mainly seen in the developing retinal vessels (23). Exposure to high levels of oxygen may lead to an increase in potentially destructive reactive oxygen species (ROS), and the return to normal oxygen environment reduces antioxidants to neutralize ROS in the retina of preterm infants, leading to endothelial cell damage, apoptosis, inflammation, and capillary constriction (24, 25).

The OIR model is commonly used to study retinal vascular diseases, including ROP because it simulates the relative hypoxia stage of ROP created by hyperoxia followed by normoxia (26, 27). Many previous reports have described miR-145-5p as a target gene of *TUG1*, and that knockdown of *TUG1* can inhibit cell migration and damage, which may have a protective effect on cells *via* targeting miR-145-5p (28–30). In our study, we found that the expression of the lncRNA *Tug1* was upregulated and the expression of miR-145-5p was down-regulated in the OIR mouse model. In our *in vitro* experiments, we induced hypoxia in HREC with CoCl_2_ to simulate the relative hypoxia state of OIR, and this is method has been used previously by Du et al. to study OIR (22). Our results also showed that when *TUG1* was knocked down or miR-145-5p was overexpressed, apoptosis, migration, and angiogenesis were inhibited, which have a protective effect on cells. Furthermore, the expression of CCN1 mRNA and protein levels were both affected, suggesting that CCN1 is regulated prior to CCN1 mRNA translation. Therefore, we propose that *TUG1* acts as a molecular sponge of miR-145-5p to regulate CCN1 expression, the dual-luciferase reporter assays also confirmed the binding relationship among them.

The changes in inflammatory and apoptotic factors are observed in many retinal diseases, including ROP (27, 31). We also found the expression of apoptotic and inflammatory factors increased and anti-apoptotic factors decreased in OIR mouse models. When we overexpressed miR-145-5p *in vivo*, retinal apoptosis and inflammation were reduced. Furthermore, it is generally believed that hypoxia leads to the increase of hypoxia-inducible factor-1 α (HIF-1α) and VEGF, which leads to the apoptosis and migration of endothelial cells resulting in RNV (32). In our study, HIF-1α and VEGF were also significantly altered in OIR mice, and overexpressing of miR-145-5p *in vivo*, decreased retinal apoptosis and neovascularization significantly. These results indicate that miR-145-5p overexpression has a protective effect on RNV/OIR. Moreover, we have previously demonstrated that knocking down TUG1 can reduce retinal apoptosis, inflammation, and damage in OIR mice (33). In conclusion, *TUG1* acts as a molecular sponge of miR-145-5p to regulate CCN1 expression and TUG1/miR-145-5p/CCN1 together are involved in the occurrence and development of RNV in OIR mice.

The current clinical treatment for ROP is mainly vitreous injection. However, the effects of vitreous injections are short-lived and most patients may require multiple injections to achieve the effect of treatment, which increases the risk of infection. Therefore, it is essential to study alternative target molecules. One of the advantages of gene therapy is that it can shorten the treatment time and work continuously (34, 35). Combined with our previous and current studies, it can be seen that a single knockdown of TUG1 or overexpression of miR-145-5p *in vivo* can achieve good therapeutic effects. Our findings reveal gene therapy as a potential long-term treatment for ROP and provide some new insights for gene therapy of neovascularization diseases. However, gene regulation is complex, and gene therapy technology as clinical treatment is not very mature, therefore, the safety of injection into the eye and the feasibility of gene therapy as a clinical application needs further research. However, we believe that with more research, gene therapy will solve not only ROP but also many other medical conditions.

## Data Availability Statement

The original contributions presented in the study are included in the article/Supplementary Material, further inquiries can be directed to the corresponding author.

## Ethics Statement

The animal study was reviewed and approved by the Medical Ethics Committee of Shengjing Hospital of China Medical University. This committee approved the use of animals for the experiments (ethics code: 2018PS239K) on 1 March 2018.

## Author Contributions

YxW: conceptualization, methodology, formal analysis, and writing—original draft preparation. YeW: data curation and visualization. XW: investigation and software. YM: resources and validation. ZL: writing—reviewing and editing. YD: funding acquisition and supervision. All authors contributed to the article and approved the submitted version.

## Conflict of Interest

The authors declare that the research was conducted in the absence of any commercial or financial relationships that could be construed as a potential conflict of interest.

## Publisher’s Note

All claims expressed in this article are solely those of the authors and do not necessarily represent those of their affiliated organizations, or those of the publisher, the editors and the reviewers. Any product that may be evaluated in this article, or claim that may be made by its manufacturer, is not guaranteed or endorsed by the publisher.

## Footnotes

1
http://starbase.sysu.edu.cn/
